# Next-Generation Sequencing of Two Mitochondrial Genomes from Family Pompilidae (Hymenoptera: Vespoidea) Reveal Novel Patterns of Gene Arrangement

**DOI:** 10.3390/ijms17101641

**Published:** 2016-10-11

**Authors:** Peng-Yan Chen, Bo-Ying Zheng, Jing-Xian Liu, Shu-Jun Wei

**Affiliations:** 1 Institute of Plant and Environmental Protection, Beijing Academy of Agriculture and Forestry Sciences, Beijing 100097, China; pengyan0508@163.com (P.-Y.C.); zhengboyingg@sina.com (B.-Y.Z.); 2 Department of Entomology, South China Agricultural University, Guangzhou 510640, China

**Keywords:** mitochondrial genomes, gene rearrangement, Vespoidea, Pompilidae, *Auplopus*, *Agenioideu*

## Abstract

Animal mitochondrial genomes have provided large and diverse datasets for evolutionary studies. Here, the first two representative mitochondrial genomes from the family Pompilidae (Hymenoptera: Vespoidea) were determined using next-generation sequencing. The sequenced region of these two mitochondrial genomes from the species *Auplopus* sp. and *Agenioideus* sp. was 16,746 bp long with an A + T content of 83.12% and 16,596 bp long with an A + T content of 78.64%, respectively. In both species, all of the 37 typical mitochondrial genes were determined. The secondary structure of tRNA genes and rRNA genes were predicted and compared with those of other insects. Atypical *trnS1* using abnormal anticodons TCT and lacking D-stem pairings was identified. There were 49 helices belonging to six domains in *rrnL* and 30 helices belonging to three domains in *rrns* present. Compared with the ancestral organization, four and two tRNA genes were rearranged in mitochondrial genomes of *Auplopus* and *Agenioideus*, respectively. In both species, *trnM* was shuffled upstream of the *trnI*-*trnQ*-*trnM* cluster, and *trnA* was translocated from the cluster *trnA*-*trnR*-*trnN*-*trnS1*-*trnE*-*trnF* to the region between *nad1* and *trnL1*, which is novel to the Vespoidea. In *Auplopus*, the tRNA cluster *trnW*-*trn*C-*trnY* was shuffled to *trnW*-*trnY*-*trnC*. Phylogenetic analysis within Vespoidea revealed that Pompilidae and Mutillidae formed a sister lineage, and then sistered Formicidae. The genomes presented in this study have enriched the knowledge base of molecular markers, which is valuable in respect to studies about the gene rearrangement mechanism, genomic evolutionary processes and phylogeny of Hymenoptera.

## 1. Introduction

Animal mitochondrial genomes are typically compact and double-stranded circular molecules of approximately 16 kb, encoding 37 genes and an A + T-rich region [1,2,3]. In addition, the mitochondrial genomes show the predominance of maternal inheritance [4,5], rare recombination [1], extremely high A + T content [6], conserved gene components [7] and relatively rapid rates of nucleotide substitution [8,9]. Therefore, the mitochondrial genomes are considered as ideal molecular markers for population genetics, species identification, as well as phylogenetic and evolutionary studies. In Hymenoptera, sequences of mitochondrial genomes have accumulated rapidly; however, representation is seriously deficient relative to the diversity of the groups. There is now sufficient data available from Hymenoptera to reliably draw conclusions about patterns and trends in the mitochondrial genome evolution of Hymenoptera.

Gene rearrangement events provide insights into investigating the dynamics of mitochondrial genomes and evolutionary relationships [10,11]. With the increasing availability of mitochondrial genomes under technical feasibility and the adoption of next-generation sequencing technologies [12,13,14,15,16], comparative study has become popular. Several orders of insect have been found exhibiting diagnostic rearrangements for major taxonomic groups [17,18,19,20]. In Hymenoptera, large-scale and complicated gene rearrangements have been found [21,22,23,24,25]. In the suborder “Symphyta”, gene rearrangement was conserved, but was accelerated in the Apocrita [10]. The rearrangement of a protein-coding gene is rare compared to the high frequency of tRNA rearrangement in Hymenoptera [22]. Most comparative studies in Hymenoptera are conducted at the superfamily level l [21,22,25,26], or at lower levels within limited groups [23,27]. Adding more mitochondrial genomes from representative groups by dense sampling will contribute to the understanding of genome evolution as well as the phylogeny of Hymenoptera.

The Pompilidae is a species-rich and cosmopolitan family belonging to the superfamily Vespoidea of Aculeata in Hymenoptera [28,29]. The pompilid species are commonly known as spider wasps or pompilid wasps [30]. For feeding their larvae, these wasps usually hunt and kill spiders often larger than themselves [31]. Wasps in Pompilidae are solitary and nest alone, which differ from many other families of Hymenoptera [32]. Most spider wasps capture and paralyze prey, though some exhibit parasitic behaviours [33]. Adult Pompilidae engage in nectar-feeding activity and feed on a variety of plants [32]. Currently, nearly complete mitochondrial genomes from Vespidae, Eumenidae, Formicidae and Mutillidae have been sequenced respectively within Vespoidea. In the sequenced mitochondrial genomes of Vespoidea, tRNA gene rearrangement was reported; however, rearrangement of protein-coding genes has not been found [22,34,35,36]. A locus of mitochondrial genes comprising *cox1* and adjacent tRNA genes was sequenced from representatives of the major clades of Pompilidae and the rearrangement of the *trnC* and *trnY* genes was found in the sequenced segments [37]. However, no complete mitochondrial genome from Pompilidae has been reported. We presumed a moderate amount of mitochondrial gene rearrangement occurred in species of Pompilidae according to current knowledge of its related families from Vespoidea [22,34,35,36], but the contribution of the rearrangement signal to phylogenetic analysis of Hymenoptera still needs confirmation.

In this study, we sequenced two mitochondrial genomes from different genera of Pompilidae, *Auplopus* and *Agenioideus*, and compared them with others across the Vespoidea. This work provides a first report of mitochondrial genomes from Pompilidae, and reveals novel gene rearrangement patterns in Vespoidea.

## 2. Results and Discussion

### 2.1. General Features of Mitochondrial Genomes

Two nearly complete mitochondrial genomes from *Auplopus* sp. (GenBank accession KX584357) and *Agenioideus* sp. (GenBank accession KX584356) were sequenced. Each genome contained all 37 typical animal mitochondrial genes, including 13 protein-coding genes, 22 tRNA genes and two rRNA genes [1,2]. The average coverage of the *Auplopus* and *Agenioideus* mitochondrial genome was 479X and 1595X, respectively, which is high compared to that of other mitochondrial genomes sequenced by using next-generation sequencing [15,38]. The complete A + T-rich region was unable to be sequenced in both species. The failure in sequencing of the A + T-rich region was common in mitochondrial genome sequencing by PCR-based method [18,21,39,40], which might be caused by the presence of the PolyA/T structure, repeat region and especially high A + T content in this region. The A + T-rich region was also difficult to determine through next-generation sequencing [15,41], possibly due to the failure of assembly from short reads (250 bp in pair-ends) rather than incomplete library construction.

For the *Auplopus* mitochondrial genome, the sequenced region was 16,746 bp long. A total of 20 bp of overlapping nucleotides were detected between genes with a length from 1 to 8 bp. A total of 551 bp of intergenic nucleotides ranging from 1 to 304 bp were found in 17 locations. In addition, there was an un-amplified portion located between *nad1* and *trnA*. Two noncoding regions with a length of 988 bp after *nad1* and 576 bp before *trnA* were present. The other eleven pairs of genes were directly adjacent, without overlapping or intergenic nucleotides.

The sequenced length of the mitochondrial genome of *Agenioideus* was 16,596 bp. In total, there were 16 bp overlapping regions in five locations (*trnI-trnQ*, *nad2-trnW*, *trnN-trnS1*, *nad4-nad4l* and *cob-trn*-*trnS2*). The shared nucleotides ranged from 2 to 6 bp, with the longest one (6 bp) located between *nad4* and *nad4l*. In total, there were 1850 bp intergenic spacer sequences in 20 locations with a length from 1 to 1240 bp. The longest non-coding region was located between *trnA* and *trnL1*. The other ten pairs of genes were directly adjacent to each other. In both species, the length of the mitochondrial genome and overlapping regions between genes was normal, while the intergenic spacer is considerably longer than other Vespoidea mitochondrial genomes [22,34,35,36].

### 2.2. Nucleotide Composition

Three parameters, AT-skew, GC-skew and A + T content, are frequently used to reveal the nucleotide-compositional behavior of mitochondrial genomes [42,43]. The sequence of whole mitochondrial genome for *Auplopus* and *Agenioideus* was biased in nucleotide composition ((A + T)% > (G + C)%) in the majority strand (J-strand), which was consistent with that of other insects. The A + T content of whole genome was 83.12% for *Auplopus* (39.04% A, 44.08% T, 8.47% G and 8.41% C), and 78.64% for *Agenioideus* (35.65% A, 42.99% T, 12.04% G and 9.32% C) (Table 1).

The A + T content of all protein-coding genes in Vespoidea ranged from 67.41% (*Leptomyrmex pallens*) to 83.38% (*Polistes jokahamae*) (Table 1). All of the AT-skews were negative, while most GC-skews were negative in Vespoidea, which indicated that the protein-coding genes contained more T and C nucleotides than A and G nucleotides, as reported for most other insects [42,43].

### 2.3. Protein-Coding Genes

Both in *Auplopus* and *Agenioideus* mitochondrial genomes, 9 of 13 protein-coding genes were located on the majority strand, while the other four protein-coding genes were located on the minority strand (N-strand). In the mitochondrial genome of *Auplopus*, the total length of protein-coding genes was 10,931 bp, accounting for 65.28% of the whole genome. The total length of the protein-coding genes of *Agenioideus* was 11,238 bp, accounting for 67.72% of the whole genome. The overall A + T content of the 13 protein-coding genes was 82.33% in *Auplopus* mitochondrial genome, ranging from 75.43% (*cox1*) to 91.82% (*atp8*) for an individual gene. In *Agenioideus* mitochondrial genome, the total A + T content of the 13 protein-coding genes was 77.72%, ranging from 71.77% (*cox1*) to 85.20% (*atp8*) for an individual gene (Table 2).

In both mitochondrial genomes, all of the protein-coding genes start with the conventional initiation codons (ATN) [44,45]. In *Auplopus*, five genes use ATA, seven use ATT and one use ATG, while in *Agenioideus*, there were three, five and five protein-coding genes starting with ATA, ATT and ATG, respectively. In *Auplopus* mitochondrial genome, 10 of 13 protein-coding genes used TAA as the stop codon, while the *nad3*, *cob* and *nad5* genes used incomplete stop codon T. In *Agenioideus* mitochondrial genome, 8 of 13 protein-coding genes terminated with TAA; the *nad4l* and *cob* genes stopped with codon TA, and the *atp8*, *nad4* and *nad6* genes stopped with codon T. The usage of incomplete stop codons of protein-coding genes is common in invertebrate mitochondrial genomes [45,46].

Relative synonymous codon usage values in the mitochondrial genomes of *Auplopus* and *Agenioideus* reflected a significant bias towards A and T nucleotides (Table 3). In both *Auplopus* and *Agenioideus* mitochondrial genomes, Leu, Ile, Phe and Met were the four most frequent amino acids and TTA (Leu), ATT (Ile), TTT (Phe) and ATA (Met) were the most frequently used codons, which was same as that in other species of Hymenoptera [6,22,27,47]. In comparison, almost all of the frequently used codons ended with A/T, which may lead to the A and T bias in the mitochondrial genome. In the mitochondrial genome of *Auplopus*, the codon Leu (CUC, CUG), Asp (GAC), Arg (CGC) and Gly (GGC) were missing, while the Arg (CGC) was absent in the mitochondrial genome of *Agenioideus*. It is obvious that the missing codons all preferred G and C in the third codon position, as those in other hymenopterans [26].

### 2.4. Transfer RNA Gene

The orientation and anticodons of the predicted tRNA genes were identical in both species. As for 22 tRNA genes detected in each mitochondrial genome, 14 genes were coded on the J-strand while eight were coded on the N-strand. In the mitochondrial genome of *Auplopus*, the tRNA genes ranged in size from 57 bp (*trnS1*) to 72 bp (*trnK*), while that in *Agenioideus* ranged from 57 bp (*trnS1*) to 69 bp (*trnK*, *trnG*). The length of tRNA usually affected the size of variable loop and D-loop regions. In the *Auplopus* and *Agenioideus*, tRNA genes had variable loops ranging from 2 to 4 bp.

All tRNA genes of the two species folded into a canonical clover-leaf structure with the dihydrouridine arm formed a simple loop, except that *trnS1* lost D-stem pairings in the DHU arm (Figures S1 and S2). The feature was the same as observed in many other insect mitochondrial genomes such as mosquito, beetle and honeybee [20,38,44,45,48,49,50,51]. There were nine wobble G–U pairs in the stem structures of *Auplopus*. There were 18 mismatches, including 15 G–U pairs, two U–U pairs and one A–A pair present in 22 tRNA genes of *Agenioideus*. Compared with other insects, the mismatches were normal in the tRNA secondary structures. The anticodons of most tRNA genes were identical to their counterparts among most other published insect mitochondrial genomes. However, the *trnS1* gene used abnormal anticodon TCT, which have been found to be correlated with frequent gene rearrangement events [38].

### 2.5. Ribosomal RNA Genes

In the mitochondrial genome of *Auplopus* and *Agenioideus*, the arrangement of both *rrnL* and *rrnS* was conserved. The position of rRNA genes was identical in both species with *rrnL* located between *trnL1* and *trnV*, and *rrnS* located downstream of *trnV*. In *Auplopus*, the *rrnL* was 1298 bp long with an A + T content of 86.06%, while the *rrnS* was 754 bp long with an A + T content of 84.62%. In *Agenioideus*, the *rrnL* had a length of 1290 bp with an A + T content of 81.16%, whereas the *rrnS* had a length of 843 bp with an A + T content of 81.02%. The length of the *rrnL* as well as *rrnS* genes was normal, and their A + T content was similar to their homolog genes in other hymenopteran insects [27,51].

The *Auplopus* and *Agenioideus* shared similar features of *rrnL* and *rrnS*. There were 49 helices present in the *rrnL* of both species, belonging to six domains (Figures S3 and S4), just as those in *Diadegma semiclausum* [6] and *Apis mellifera* [52]. The predicted structures of helix 837 usually form a long stem structure with a small loop in the terminal [20,52], but it formed a shorter stem and a larger loop in the two newly sequenced species which conformed to that in *Evania appendigaster* [38] and *D. melanogaster*. H991 displayed helical length and loop size variability between *Auplopus* and *Agenioideus*. H2520 was variable in length and shape in *Auplopus* and *Agenioideus* as in other insects [53,54].

There were 30 helices found in *rrnS* of *Auplopus* and *Agenioideus* belonging to three domains as reported in braconid species [22] and *E. appendigaster* [38]. H39 was not predicted in *Auplopus* and *Agenioideus*, instead of a circle formed by H27, H47, H367 and H500, and the sequences in between [38] (Figures S5 and S6). Loop number and size variability of helix 47 were commonly observed features in *rrnS* of Hymenoptera. In *Auplopus* and *Agenioideus*, H47 formed two loops that were similar to *E. appendigaster* [38] and *D. virilis* [55], but different from that in *D. semiclausum* [6] and *A. mellifera* [52] where a larger loop was present, and that in the *Cephus* species with four loops in the same position [56]. Contrastingly, H673 was conserved in *Auplopus* and *Agenioideus*, which wassimilar to *D. virilis* [55] and *D. Semiclausum* [6].

### 2.6. Gene Rearrangement

**Figure 1:**
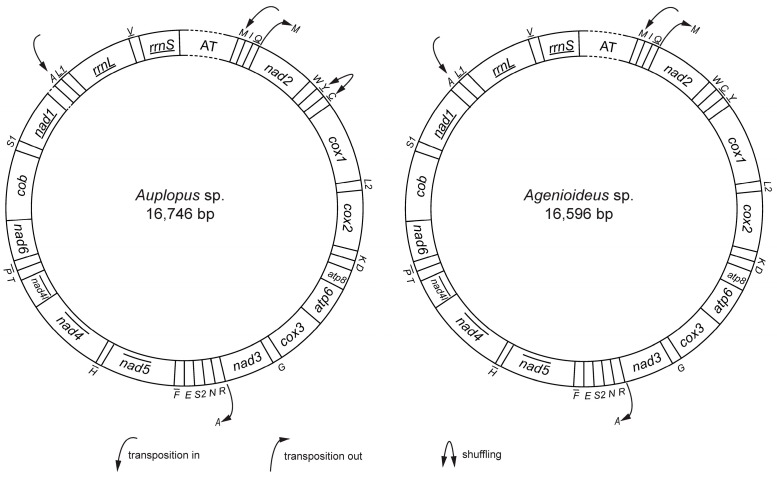
Map and rearrangement of the mitochondrial genomes in *Agenioideus* and *Auplopus*. Genes with underscores are encoded on the minority strand. Dashed lines indicate the unsequenced region of the genome. *cox1*, *cox2*, and *cox3*: cytochrome oxidase subunits; *cob*: cytochrome b; *nad1*-*nad6*: NADH dehydrogenase components; *rrnL* and *rrnS*: ribosomal RNAs. One-letter symbol refers to the transfer RNA gene according to the IPUC-IUB single-letter amino acid codes. L1, L2, S1 and S2: *tRNA^Leu(CUN)^*, *tRNA^Leu(UUR)^*, *tRNA^Ser(AGN)^*, and *tRNA^Ser(UCN)^*.

In the mitochondrial genome of *Auplopus* and *Agenioideus*, protein-coding and rRNA genes displayed the same order and orientation as those present in the putative ancestral mitochondrial genome of insect [57,58,59] (Figure 1). However, four and two tRNA genes were rearranged in *Auplopus* and *Agenioideus*, respectively.

Gene rearrangement events have been classified into transposition (translocation), local inversions (inverted in the local position), gene shuffling (local translocation) and remote inversions (translocated and inverted) [60]. The *trnM* gene was shuffled upstream of the *trnI*-*trnQ*-*trnM* cluster applying to *Auplopus* and *Agenioideus*, which might be explained by the TDRL model (tandem duplication followed by random loss) with the evidence that in *Auplopus*, a 6 bp, and in *Agenioideus,* a 156 bp intergenic region was found between *trnQ* and *nad2* as described in *E. appendigaster* [38]. Evidence for the TDRL mechanism is indicated by the pattern of gene order, the presence of pseudogenes or duplicated genes, and the position of intergenic spacers [22]. The intergenic spacer between *trnQ* and *nad2* may be a remnant region after deletion of the secondary copy of *trnM*. In both species, *trnA* was translocated from the *trnA*-*trnR*-*trnN*-*trnS1*-*trnE*-*trnF* cluster to the location between *nad1* and *trnL1*, which is a novel arrangement pattern within the Vespoidea. The duplication/random loss model, and the intramitochondrial genome recombination [61,62,63] and duplication/nonrandom loss [64] model are possible mechanisms to explain translocation [22]. The shuffling of *trnY* and *trnC* genes among the cluster *trnW*-*trnC*-*trnY* occurred in *Auplopus* but not *Agenioideus*; the rearrangement event could also be found in *Psorthaspis legata*, *Ageniella agenioides* and *Calopompilus maculipennis* of Pompilidae [37]*.*

In the Aculeata, protein-coding gene rearrangement has been found in the *Cephalonomia* mitochondrial genome [22]. However, there was no protein-coding gene rearrangement detected in Vespoidea, which also applied to the Pompilidae according to the mitochondrial genomes first reported here. Rearrangement of the tRNA gene is a typical feature of the mitochondrial architecture in Hymenoptera [17,21,40]. In Vespoidea, various extents of tRNA gene rearrangement has been found. Comparisons of the mitochondrial genomes within Vespoidea revealed similar patterns of gene arrangements in some tRNA genes. Most species formed the identical arrangement pattern of *trnM*-*trnI*-*trnQ*, but *Wallacidia oculata* (Mutillidae), *Camponotus atrox* (Formicidae) [65], *Vespa bicolo*r (Vespidae) [66], and *Vespa mandarinia* (Vespidae) [35] followed an arrangement pattern of *trnI*–*trnM*-*trnQ*. However, the remote transposition pattern in the Pompilidae presented in this study has not been reported in Vespoidea. Rearrangements in the *trnA*-*trnR*-*trnN*-*trnS1*-*trnE*-*trnF* cluster rarely occurred in the Vespoidea mitochondrial genomes previously reported. The *trnN* translocation to the downstream of *rrnS* in *Solenopsis geminata* (Formicidae) and *Solenopsis invicta* [67] and translocation to the upstream of *trnM*-*trnI*-*trnQ* in *Linepithema* *humile* (Formicidae) [68] was reported. Our analyses indicate that gene rearrangements in Vespoidea are randomly distributed but may be conserved within genus, such as the *Solenopsis* [67].

### 2.7. Phylogenetic Relationships

**Figure 2:**
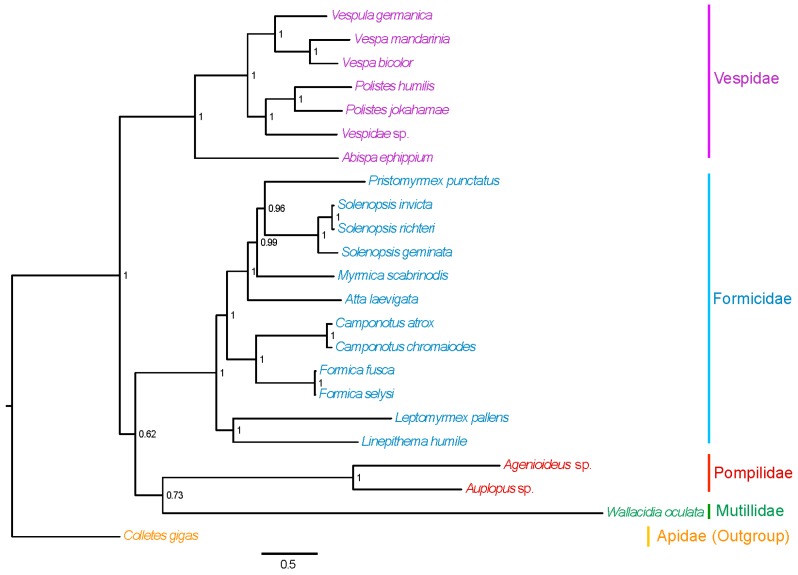
Bayesian phylogenetic tree of the superfamily Vespoidea of Hymenoptera based on the mitochondrial genome sequences. The number indicates the posterior probability of the corresponding node nearby.

Phylogenetic relationships within the superfamily Vespoidea were reconstructed (Figure 2). The result supported the monophyly of Vespidae, as revealed by previous studies [33,34,35]. Among the currently used species of Vespoidea, the Pompilidae and Mutillidae formed a sister lineage, congruent with a previous study [69]. This is the first time that the mitochondrial genomes of Pompilidae were used to investigate the phylogenetic relationships within Vespoidea [21,22,25]. Extensive sequencing of the mitochondrial genomes from other relative species is needed to reveal the phylogenetic relationships within Vespoidea.

## 3. Materials and Methods

### 3.1. Sample Collection and DNA Extraction

The specimen of *Auplopus* sp. was collected from Tianmu Mountain of Zhejiang Province, China, July 2015, and identified by Akira Shimizu. While the specimen of *Agenioideus* sp. was collected from the Haidian district of Beijing, China, June 2015, and identified by Hua-Yan Chen. Both specimens were stored at −80 °C in 100% ethanol prior to DNA extraction. Total genomic DNA was extracted separately from legs and thorax of each individual specimen with the DNeasy tissue kit (Qiagen, Hilden, Germany) following the manufacturer’s protocol. Voucher DNA was deposited in the entomological collections of Beijing Academy of Agriculture and Forestry Sciences.

### 3.2. Mitochondrial Genome Sequencing and Assembly

The mitochondrial genome sequences were gained by next-generation sequencing. Prior to library construction, the DNA was quantified by Qubit 3.0 (Invitrogen, Life technologies, Carlsbad, CA, USA). The library with two indexes was constructed using the Illumina TruSeq@ DNA PCR-Free HT Kit and sequenced by BerryGenomics Company (Beijing, China) using Illumina Miseq 2500 with the strategy of 250 paired-ends.

The mitochondrial data constitutes a small fraction of huge primary data generated by genomic sequencing (approximately 0.5%) [11,47]. To simplify the de novo assembly of mitochondrial genome from short reads produced, the mitochondrial targets were filtered at the stage of raw reads by similarity searches against a database of hymenoptera mitochondrial genomes, using BLASTn version 2.2.27+ with the *E* value of 1 × 10^−5^ and maximum target sequences of 1. Putative mitochondrial reads allowing for blast hits were extracted with a Perl script (FastqExtract.pl) [41]. All putative mitochondrial reads from the library were assembled into contigs with Celera Assembler version 8.3rc2 and IDBA version 1.1.1 as described in [15]. The de novo assembly of the mitochondrial contigs generated in previous methods were conducted by Geneious version 9.1.4 [70].

### 3.3. Mitochondrial Genome Annotation

The initial identification and annotation of the genome was conducted by Mitos WebServer [71] with the genetic code of Invertebrate Mitochondria. The boundaries of protein-coding genes were examined again by alignment against their homologs in the Vespoidea. Putative tRNA genes were identified using the tRNAscan-SE search server with a Cove cutoff score of 5. When expected tRNA genes could not be found, alignment of candidate regions was conducted with the homologous genes in relative species. The gene boundaries of rRNA genes and control region were assigned based on alignment with their homologs and the ends of neighboring tRNAs.

### 3.4. Comparative Analysis of the Mitochondrial Genomes

A total of 22 species from Vespoidea were involved in analysis, including 12 species of Formicidae, seven species of Vespidae, two species of Pompilidae and one species of Mutillidae, whose mitochondrial genomes were sequenced (Table 4). We analyzed the features of mitochondrial genomes including nucleotide composition, codon usage, nucleotide diversity and gene arrangement. The nucleotide composition was calculated by MEGA5 [72]. The AT and GC asymmetries, called AT-skews and GC-skews, were calculated based on formula AT-skew = (A% − T%)/(A% + T%) and GC-skew = (G% − C%)/(G% + C%) [73]. The Relative Synonymous Codon Usage (RSCU) of all protein-coding genes was analyzed in codonW (written by John Peden, University of Nottingham, Nottingham, UK).

The secondary structures of both *rrnL* and *rrnS* were predicted by comparative sequence method using XRNA version (developed by B. Weiser and available online: http://rna.ucsc.edu/rnacenter/xrna/xrna.html) [74]. Secondary structures of the tRNA genes were predicted using the tRNAscan-SE search server [75] and re-drawn by using XRNA.

Gene order of the two newly sequenced mitochondrial genomes were compared with the putative ancestral arrangement of insect mitochondrial genome [9] as well as all currently sequenced mitochondrial genomes in Vespoidea.

### 3.5. Phylognetic Analysis

To investigate the phylogenetic relationships within the Vespoidea, 22 species from the Vespoidea (Table 4) were included. The phylogenetic tree was reconstructed with the Bayesian inference method (BI) using the MrBayes version 3.2.5 [76] based on the nucleotide sequences of the 13 protein-coding genes. The sequences were aligned using the MAFFT version 7.205 [77]. The best schemes of partition and substitution models (Table 5) were determined by the PartitionFinder version 1.1.1 [78]. Four independent Markov chains were run for 10 million metropolis-coupled generations, with tree sampling occurring every 1000 generations and a burn-in of 25% trees. The *Colletes gigas* from the superfamily Apoidea was used as outgroup [79].

## 4. Conclusions

We sequenced and characterized the mitochondrial genomes of *Auplopus* sp. and *Agenioideus* sp., the first two representatives from the Pompilidae, using next-generation sequencing. The mitochondrial genome organization and gene rearrangements of the two species were comparatively analyzed. A novel pattern of gene rearrangement to Vespoidea was revealed in this study. Our work provides fundamental datasets for studies of gene rearrangement mechanisms and evolutionary processes and inferring of phylogenetic relationships in the Hymenoptera.

## Figures and Tables

**Table 1:** Base composition of the mitochondrial genomes in Vespoidea.

Species	Whole Genome							All Protein-Coding Genes						
	T%	C%	A%	G%	(A + T)%	AT-skew	GC-skew	T%	C%	A%	G%	(A + T)%	AT-skew	GC-skew
*Agenioideua* sp.	42.99	9.32	35.65	12.04	78.64	−0.0932	0.1272	44.95	10.66	32.76	11.62	77.72	−0.1569	0.0431
*Auplopus* sp.	44.08	8.41	39.04	8.47	83.12	−0.0607	0.0036	47.47	8.39	34.85	9.29	82.33	−0.1532	0.0507
*Wallacidia oculata*	33.58	14.56	43.78	8.08	77.36	0.1318	−0.2864	41.17	12.40	33.84	12.59	75.01	−0.0978	0.0075
*Solenopsis geminata*	37.94	16.95	38.60	6.51	76.54	0.0086	−0.4452	42.26	14.86	31.32	11.56	73.58	−0.1487	−0.1250
*Solenopsis invicta*	38.65	16.52	38.53	6.31	77.18	−0.0015	−0.4472	42.93	14.29	31.31	11.47	74.24	−0.1564	−0.1096
*Solenopsis richteri*	38.55	16.59	38.39	6.47	76.95	−0.0021	−0.4391	42.84	14.37	31.23	11.57	74.07	−0.1567	−0.1079
*Myrmica scabrinodis*	42.66	11.27	39.52	6.54	82.19	−0.0382	−0.2659	46.17	10.18	34.01	9.63	80.19	−0.1516	−0.0279
*Pristomyrmex punctatus*	40.65	14.28	38.98	6.09	79.64	−0.0210	−0.4024	43.95	11.90	33.99	10.16	77.94	−0.1278	−0.0790
*Leptomyrmex pallens*	32.60	22.01	36.94	8.45	69.54	0.0624	−0.4452	38.69	17.49	28.71	15.11	67.41	−0.1480	−0.0729
*Atta laevigata*	43.60	14.18	37.25	4.98	80.84	−0.0785	−0.4799	44.70	12.10	32.98	10.22	77.68	−0.1508	−0.0842
*Formica fusca*	43.07	10.96	40.35	5.63	83.42	−0.0326	−0.3215	46.77	9.95	34.37	8.91	81.14	−0.1528	−0.0551
*Formica selysi vouche*	42.94	11.07	40.33	5.66	83.27	−0.0313	−0.3236	46.81	10.06	34.20	8.92	81.01	−0.1556	−0.0599
*Camponotus chromaiodes*	38.77	14.93	39.37	6.93	78.14	0.0077	−0.3661	44.53	12.39	32.58	10.49	77.12	−0.1550	−0.0832
*Camponotus atrox*	39.87	14.75	38.97	6.42	78.83	−0.0114	−0.3933	44.06	12.73	32.34	10.86	76.41	−0.1534	−0.0790
*Linepithema humile*	41.27	6.23	39.05	13.45	80.32	−0.0277	0.3668	45.02	11.36	33.17	10.46	78.18	−0.1516	−0.0411
*Polistes humilis*	41.65	9.95	43.09	5.32	84.73	0.0170	−0.3031	46.61	8.51	36.77	8.11	83.38	−0.1180	−0.0244
*Polistes jokahamae*	41.45	10.79	41.97	5.80	83.41	0.0062	−0.3012	45.51	9.70	36.00	8.79	81.51	−0.1167	−0.0491
*Vespidae* sp.	39.46	11.19	43.07	6.28	82.53	0.0437	−0.2810	44.93	9.87	35.36	9.84	80.28	−0.1192	−0.0014
*Vespa bicolor*	40.98	12.81	40.74	5.47	81.72	−0.0030	−0.4012	44.32	11.11	35.00	9.57	79.31	−0.1175	−0.0745
*Abispa ephippium*	41.05	13.38	39.55	6.02	80.61	−0.0187	−0.3796	43.48	11.21	35.20	10.12	78.67	−0.1052	−0.0510
*Vespa mandarinia*	40.51	14.53	38.88	6.07	79.39	−0.0205	−0.4104	43.37	12.35	33.73	10.56	77.09	−0.1251	−0.0781
*Vespula germanica*	41.47	12.39	40.21	5.94	81.67	−0.0154	−0.3523	45.45	10.55	33.83	10.17	79.28	−0.1465	−0.0186

**Table 2:** Base composition of protein-coding and rRNA genes in the mitochondrial genomes of *Agenioideus* and *Auplopus*.

GeneSpecies	*Agenioideus* sp.							*Auplopus* sp.						
	T%	C%	A%	G%	(A + T)%	AT-skew	GC-skew	T%	C%	A%	G%	(A + T)%	AT-skew	GC-skew
*atp6*	49.57	7.98	29.20	13.25	78.77	−0.2586	0.2483	50.00	8.86	32.58	8.56	82.58	−0.2109	−0.0172
*atp8*	48.00	6.80	37.20	8.00	85.20	−0.1268	0.0811	47.80	5.03	44.03	3.14	91.82	−0.0411	−0.2308
*cob*	46.07	10.88	28.56	14.50	74.62	−0.2346	0.1429	45.08	10.89	32.57	11.46	77.65	−0.1611	0.0256
*cox1*	45.09	12.47	26.68	15.76	71.77	−0.2565	0.1167	43.95	10.94	31.48	13.63	75.43	−0.1654	0.1094
*cox2*	41.74	10.58	34.20	13.48	75.94	−0.0992	0.1205	46.09	9.42	35.22	9.28	81.30	−0.1337	−0.0078
*cox3*	48.23	10.10	27.40	14.27	75.63	−0.2755	0.1710	48.91	9.45	31.16	10.47	80.08	−0.2217	0.0513
*nad1*	43.41	12.67	34.53	9.38	77.94	−0.1140	−0.1493	48.26	7.95	33.66	10.13	81.92	−0.1782	0.1205
*nad2*	50.65	5.54	32.53	11.28	83.18	−0.2179	0.3413	50.70	5.62	36.55	7.13	87.25	−0.1623	0.1181
*nad3*	52.42	6.84	28.21	12.54	80.63	−0.3004	0.2941	56.16	4.58	30.95	8.31	87.11	−0.2895	0.2889
*nad4*	39.79	12.65	39.56	8.00	79.34	−0.0029	−0.2251	46.44	6.74	38.08	8.74	84.52	−0.0988	0.1287
*nad4l*	44.09	9.68	39.07	7.17	83.15	−0.0603	−0.1489	48.55	5.43	39.49	6.52	88.04	−0.1029	0.0909
*nad5*	40.97	13.24	37.67	8.12	78.64	−0.0421	−0.2394	46.07	8.83	37.48	7.62	83.56	−0.1027	−0.0735
*nad6*	46.96	7.03	32.89	13.12	79.85	−0.1762	0.3019	52.19	6.10	37.33	4.38	89.52	−0.1660	−0.1636
*rrnL*	37.29	8.60	43.88	10.23	81.16	0.0812	0.0864	38.44	5.78	47.61	8.17	86.06	0.1065	0.1713
*rrnS*	34.64	8.78	46.38	10.20	81.02	0.1449	0.0750	37.27	6.37	47.35	9.02	84.62	0.1191	0.1724

**Table 3:** Codon usage in the mitochondrial genomes of *Agenioideus* and *Auplopus*.

*Agenioideus* sp.												*Auplopus* sp.											
AA	Codon	No.	RSCU	AA	Codon	No.	RSCU	AA	Codon	No.	RSCU	AA	Codon	No.	RSCU	AA	Codon	No.	RSCU	AA	Codon	No.	RSCU
Phe	UUU	335	1.83	Ser	UCU	125	2.44	Tyr	UAU	163	1.71	Phe	UUU	395	1.96	Ser	UCU	107	2.32	Tyr	UAU	175	1.91
	UUC	31	0.17		UCC	17	0.33		UAC	28	0.29		UUC	9	0.04		UCC	3	0.07		UAC	8	0.09
Leu	UUA	408	4.6		UCA	115	2.24	Cys	UGU	43	1.87	Leu	UUA	502	5.68		UCA	140	3.04	Cys	UGU	41	1.95
	UUG	35	0.39		UCG	5	0.1		UGC	3	0.13		UUG	8	0.09		UCG	5	0.11		UGC	1	0.05
	CUU	35	0.39	Pro	CCU	67	2.29	His	CAU	52	1.68		CUU	17	0.19	Pro	CCU	66	2.38	His	CAU	60	1.9
	CUC	4	0.05		CCC	12	0.41		CAC	10	0.32		CUC	0	0		CCC	2	0.07		CAC	3	0.1
	CUA	48	0.54		CCA	33	1.13	Gln	CAA	43	1.79		CUA	3	0.03		CCA	40	1.44	Gln	CAA	50	1.92
	CUG	2	0.02		CCG	5	0.17		CAG	5	0.21		CUG	0	0		CCG	3	0.11		CAG	2	0.08
Ile	AUU	389	1.87	Thr	ACU	76	2.01	Asn	AAU	154	1.58	Ile	AUU	439	1.98	Thr	ACU	82	2.58	Asn	AAU	199	1.91
	AUC	26	0.13		ACC	7	0.19		AAC	41	0.42		AUC	5	0.02		ACC	3	0.09		AAC	9	0.09
Met	AUA	306	1.8		ACA	63	1.67	Lys	AAA	113	1.71	Met	AUA	315	1.9		ACA	37	1.17	Lys	AAA	121	1.92
	AUG	34	0.2		ACG	5	0.13		AAG	19	0.29		AUG	16	0.1		ACG	5	0.16		AAG	5	0.08
Val	GUU	99	2.04	Ala	GCU	55	2.22	Asp	GAU	65	1.91	Val	GUU	79	2.36	Ala	GCU	42	2.05	Asp	GAU	58	2
	GUC	6	0.12		GCC	9	0.36		GAC	3	0.09		GUC	1	0.03		GCC	3	0.15		GAC	0	0
	GUA	76	1.57		GCA	33	1.33	Glu	GAA	55	1.45		GUA	52	1.55		GCA	37	1.8	Glu	GAA	73	1.95
	GUG	13	0.27		GCG	2	0.08		GAG	21	0.55		GUG	2	0.06		GCG	0	0		GAG	2	0.05
Gly	GGU	57	1.56	Arg	CGU	20	1.74	Ser	AGU	48	0.94	Gly	GGU	48	1.21	Arg	CGU	20	1.7	Ser	AGU	24	0.52
	GGC	7	0.19		CGC	0	0		AGC	3	0.06		GGC	0	0		CGC	0	0		AGC	2	0.04
	GGA	47	1.29		CGA	20	1.74		AGA	80	1.56		GGA	107	2.69		CGA	26	2.21		AGA	87	1.89
	GGG	35	0.96		CGG	6	0.52		AGG	17	0.33		GGG	4	0.1		CGG	1	0.09		AGG	1	0.02
Trp	UGA	71	1.41	–	–	–	–	–	–	–	–	Trp	UGA	83	1.91	–	–	–	–	–	–	–	–
	UGG	30	0.59		–	–	–	–	–	–	–		UGG	4	0.09		–	–	–		–	–	–

RSCU: Relative Synonymous Codon Usage; AA: Amino Acid; No.: Number.

**Table 4:** The mitochondrial genomes currently sequenced in the different species of Vespoidea.

Species	Superfamily	Family	Accession Number	References
*Agenioideua* sp.	Vespoidea	Pompilidae	KX584356	This study
*Auplopus* sp.	Vespoidea	Pompilidae	KX584357	This study
*Wallacidia oculata*	Vespoidea	Mutillidae	FJ611801	[22]
*Solenopsis geminata*	Vespoidea	Formicidae	HQ215537	[67]
*Solenopsis invicta*	Vespoidea	Formicidae	HQ215538	[67]
*Solenopsis richteri*	Vespoidea	Formicidae	HQ215539	[67]
*Myrmica scabrinodis*	Vespoidea	Formicidae	LN607806	[80]
*Pristomyrmex punctatus*	Vespoidea	Formicidae	AB556946	[81]
*Leptomyrmex pallens*	Vespoidea	Formicidae	KC160533	[82]
*Atta laevigata*	Vespoidea	Formicidae	KC346251	[83]
*Formica fusca*	Vespoidea	Formicidae	LN607805	[80]
*Formica selysi*	Vespoidea	Formicidae	KP670862	[84]
*Camponotus chromaiodes*	Vespoidea	Formicidae	JX966368	[85]
*Camponotus atrox*	Vespoidea	Formicidae	KT159775	[65]
*Linepithema humile*	Vespoidea	Formicidae	KT428891	[68]
*Polistes humilis*	Vespoidea	Vespidae	EU024653	[40]
*Polistes jokahamae*	Vespoidea	Vespidae	KR052468	[35]
*Vespidae* sp.	Vespoidea	Vespidae	KM244667	[86]
*Vespa bicolor*	Vespoidea	Vespidae	KJ735511	[66]
*Abispa ephippium*	Vespoidea	Vespidae	NC011520	[40]
*Vespa mandarinia*	Vespoidea	Vespidae	KR059904	[36]
*Vespula germanica*	Vespoidea	Vespidae	KR703587	[34]

**Table 5:** The best schemes of partition and substitution models of 13 protein-coding genes in 22 species of Vespoidea.

Optimal Partition	Model	Initial Partition
Partition 1	GTR + I + G	*a6p1*, *c2p1*, *c3p1*, *cbp1*, *n3p1*
Partition 2	HKY + I + G	*a8p1*, *n2p1*, *n6p1*
Partition 3	GTR + I + G	*a6p2*, *c2p2*, *c3p2*, *cbp2*, *n3p2*
Partition 4	GTR + I + G	*n1p2*, *n4lp2*, *n4p2*, *n5p2*
Partition 5	GTR + G	*c1p2*
Partition 6	GTR + G	*a8p2*, *n2p2*, *n6p2*
Partition 7	HKY + G	*a8p3*, *n2p3*, *n6p3*
Partition 8	GTR + I + G	*n1p1*, *n4lp1*, *n4p1*, *n5p1*
Partition 9	HKY + G	*n1p3*, *n4lp3*, *n4p3*, *n5p3*
Partition 10	GTR + G	*a6p3*, *c1p3*, *c2p3*, *c3p3*, *cbp3*, *n3p3*
Partition 11	GTR + I + G	*c1p1*

The initial partitions were defined by gene and codon position. Each partition was named using the first and last letter of the gene name followed by codon position. *p1*, *p2* and *p3* in column “Initial Partition” indicates the first, second and third codon position.

## Supplementary Materials

Supplementary materials can be found at www.mdpi.com/1422-0067/17/10/1641/s1.
