# Supplementary material for: Next-Generation Sequencing of Two Mitochondrial Genomes from Family Pompilidae (Hymenoptera: Vespoidea) Reveal Novel Patterns of Gene Arrangement
Source: Int J Mol Sci. 2016 Oct 11;17(10):1641. doi: 10.3390/ijms17101641 (PMC5085674; doi:10.3390/ijms17101641)
Supplement: Supplementary file 1 [file ijms-17-01641-s001.pdf]

Supplementary Materials: Next-Generation Sequencing of Two Mitochondrial Genomes from Family Pompilidae (Hymenoptera: Vespoidea) Reveal Novel Patterns of Gene Arrangement

Peng-Yan Chen, Bo-Ying Zheng, Jing-Xian Liu and Shu-Jun Wei

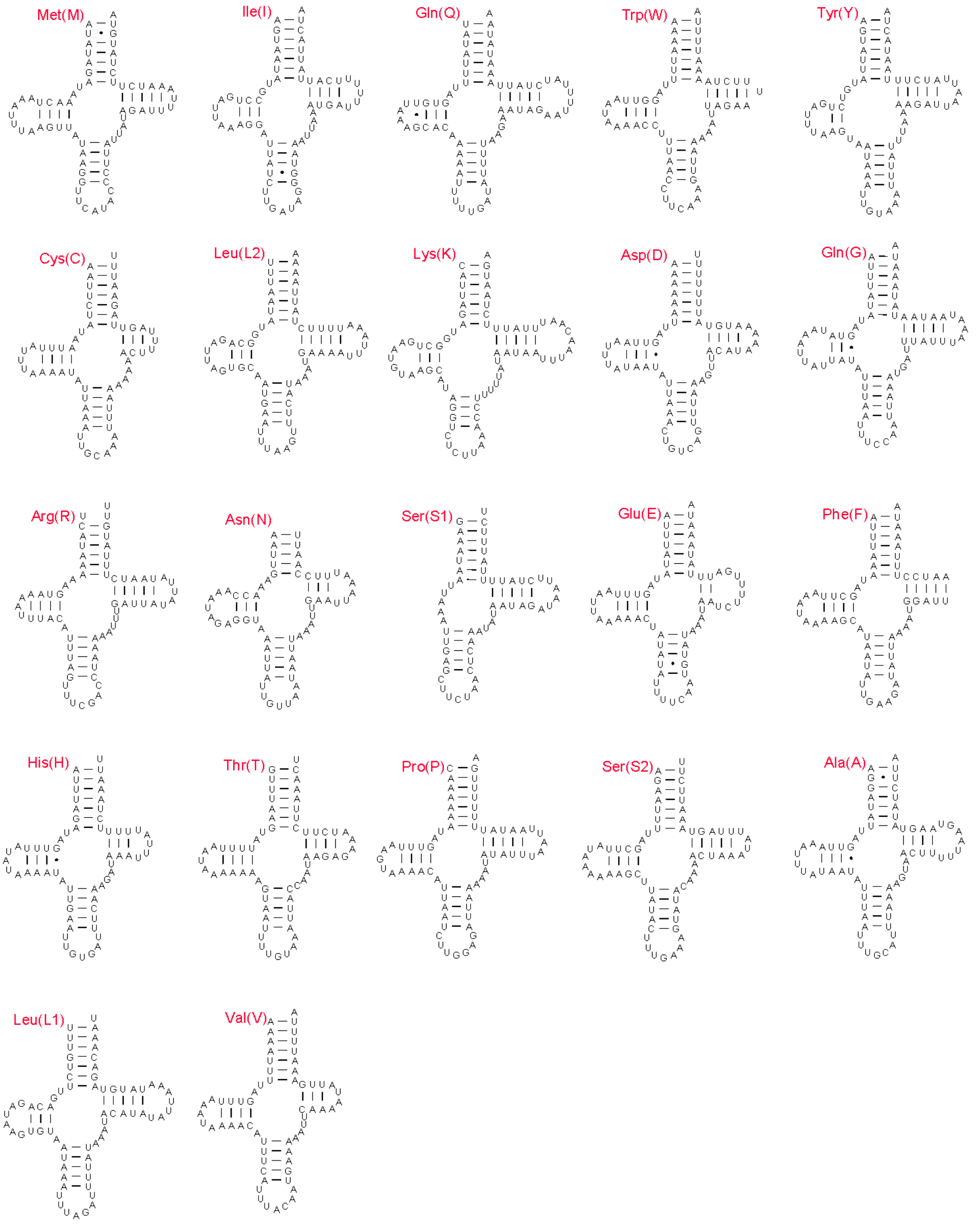

Figure S1. Predicted secondary structures for the 22 typical tRNA genes of *Auplopus* mitochondrial genome. Base-pairing is indicated as follows: Watson–Crick pairs by lines, wobble GU pairs by dots and other noncanonical pairs by circles.

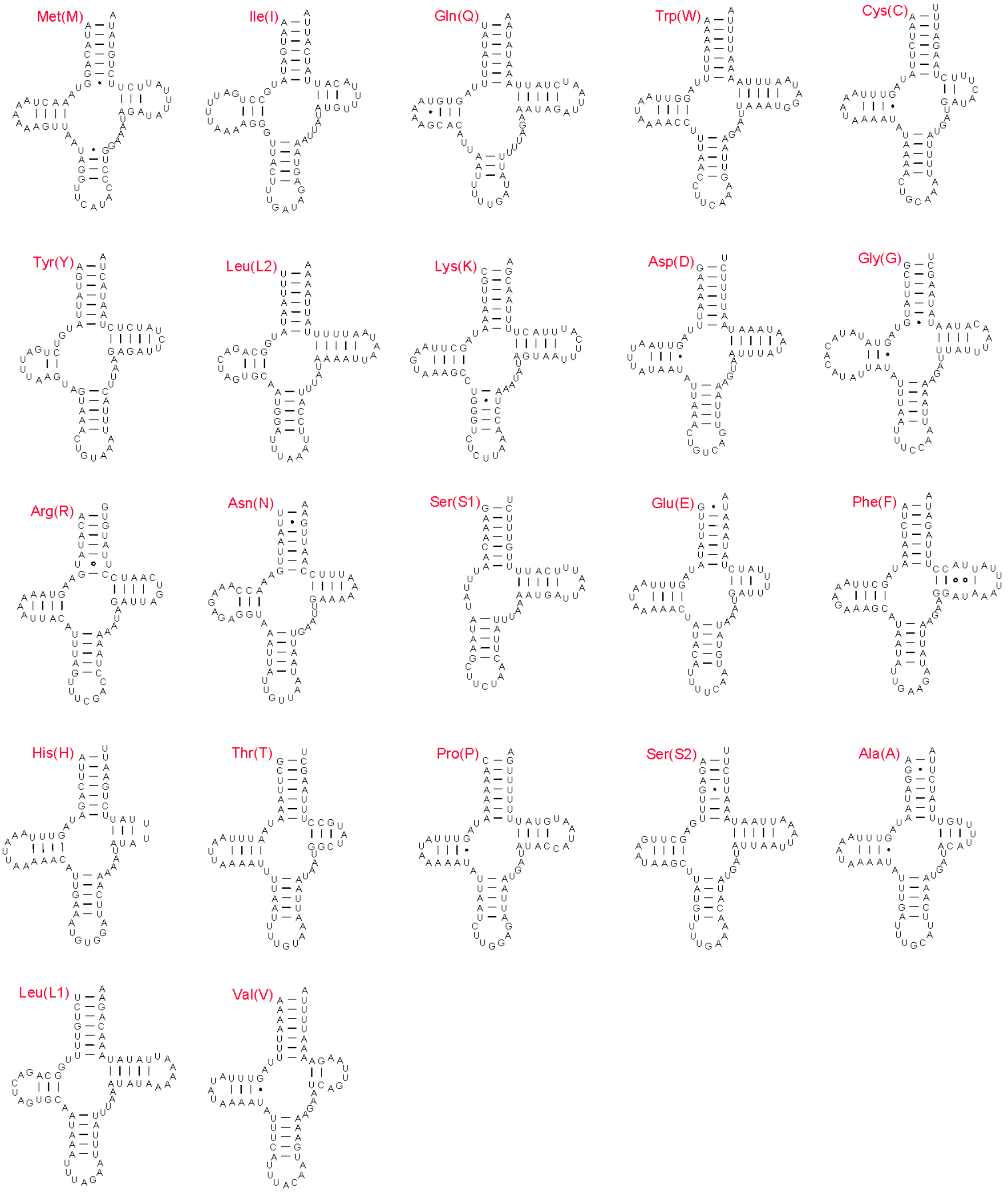

Figure S2. Predicted secondary structures for the 22 typical tRNA genes of *Agenioideus* mitochondrial genome. Symbols are as in Figure S1.

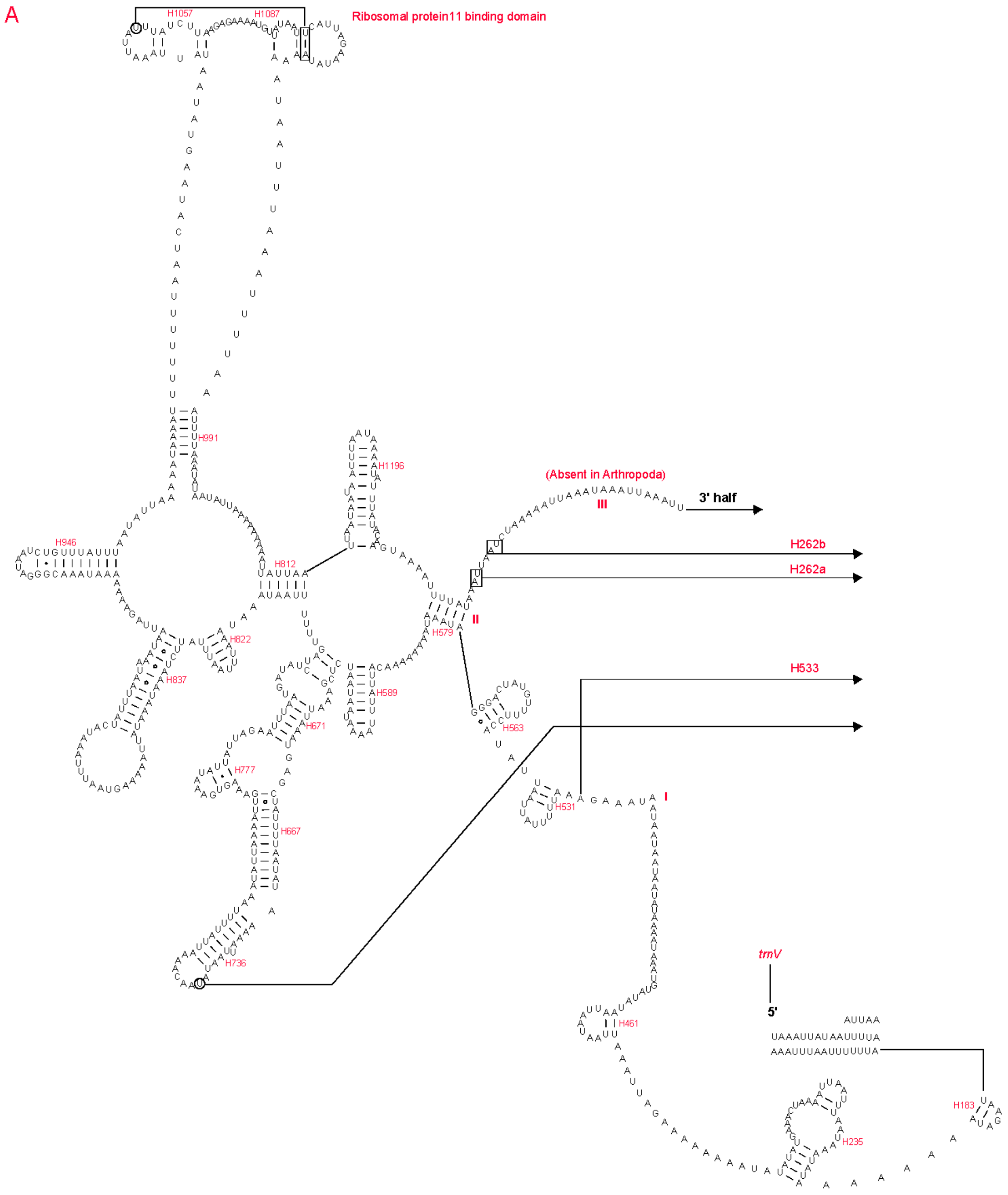

Figure S3. *Cont.*

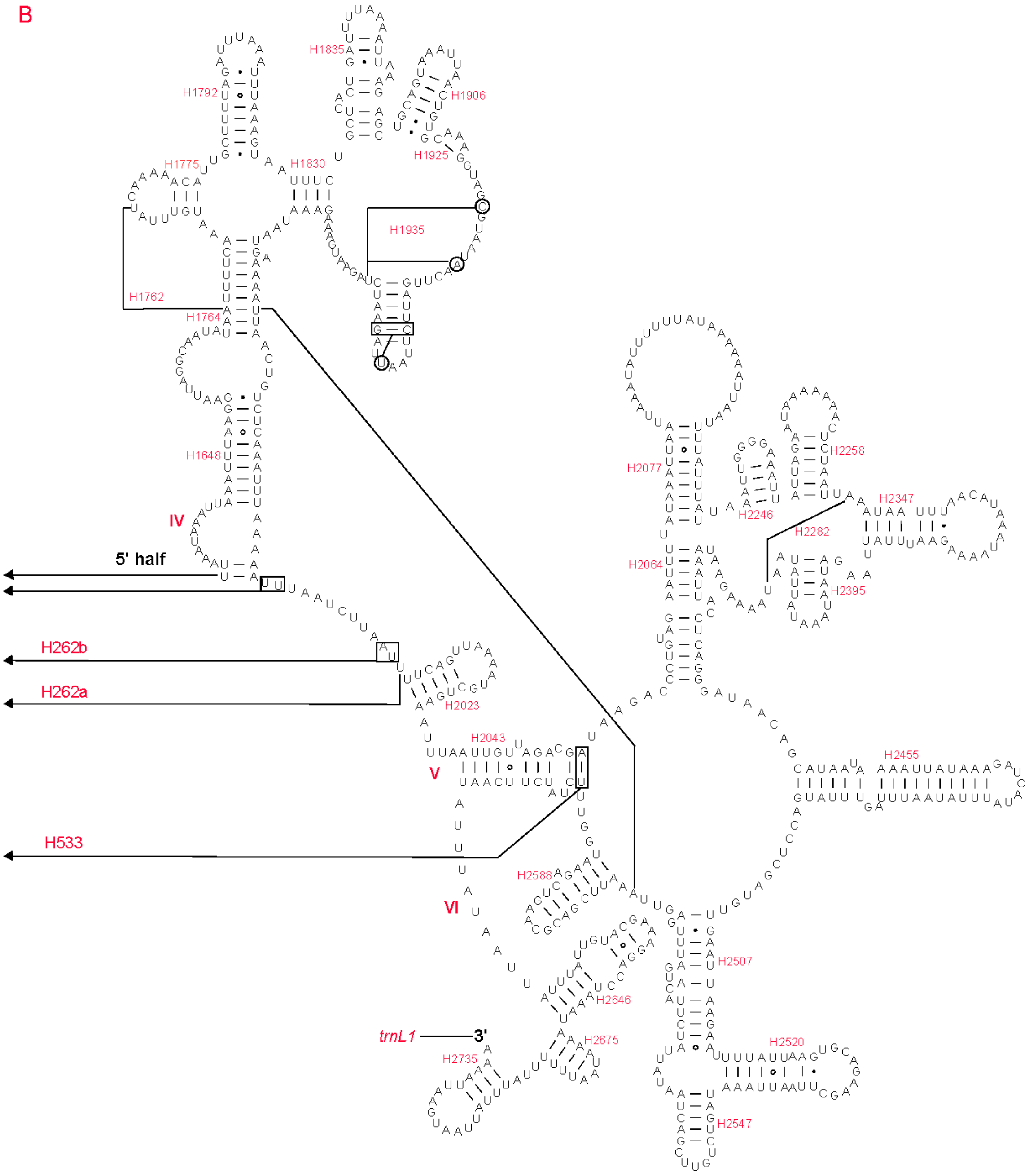

Figure S3. Predicted *rrnL* secondary structure in the *Auplopus* mitochondrial genome. Tertiary interactions and base triples are connected by continuous lines. (**A**) 5′ half of *rrnL*; (**B**) 3′ half of *rrnL*. Symbols are as in Figure S1. I–VI shows the region of different domains.

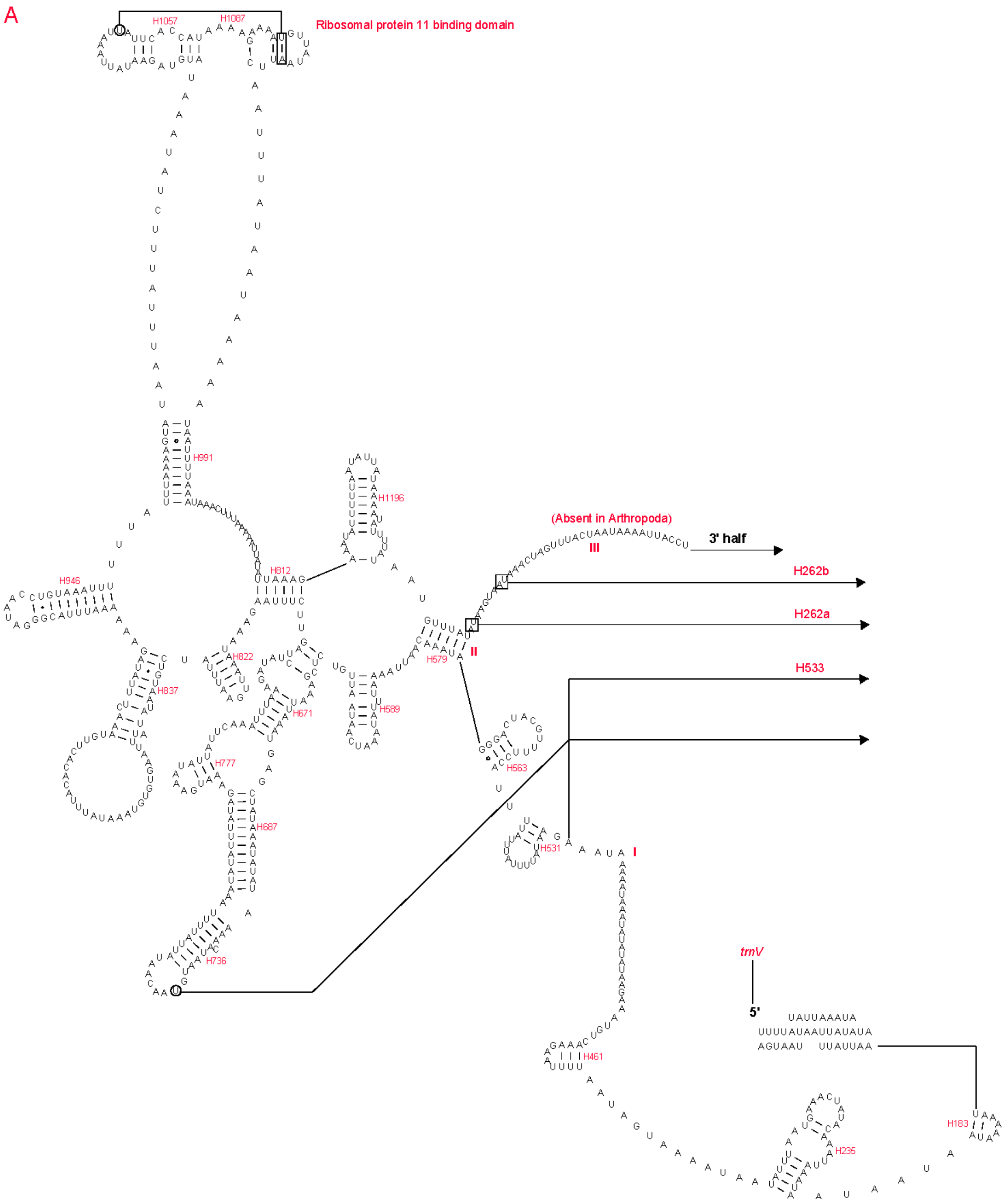

Figure S4. *Cont.*

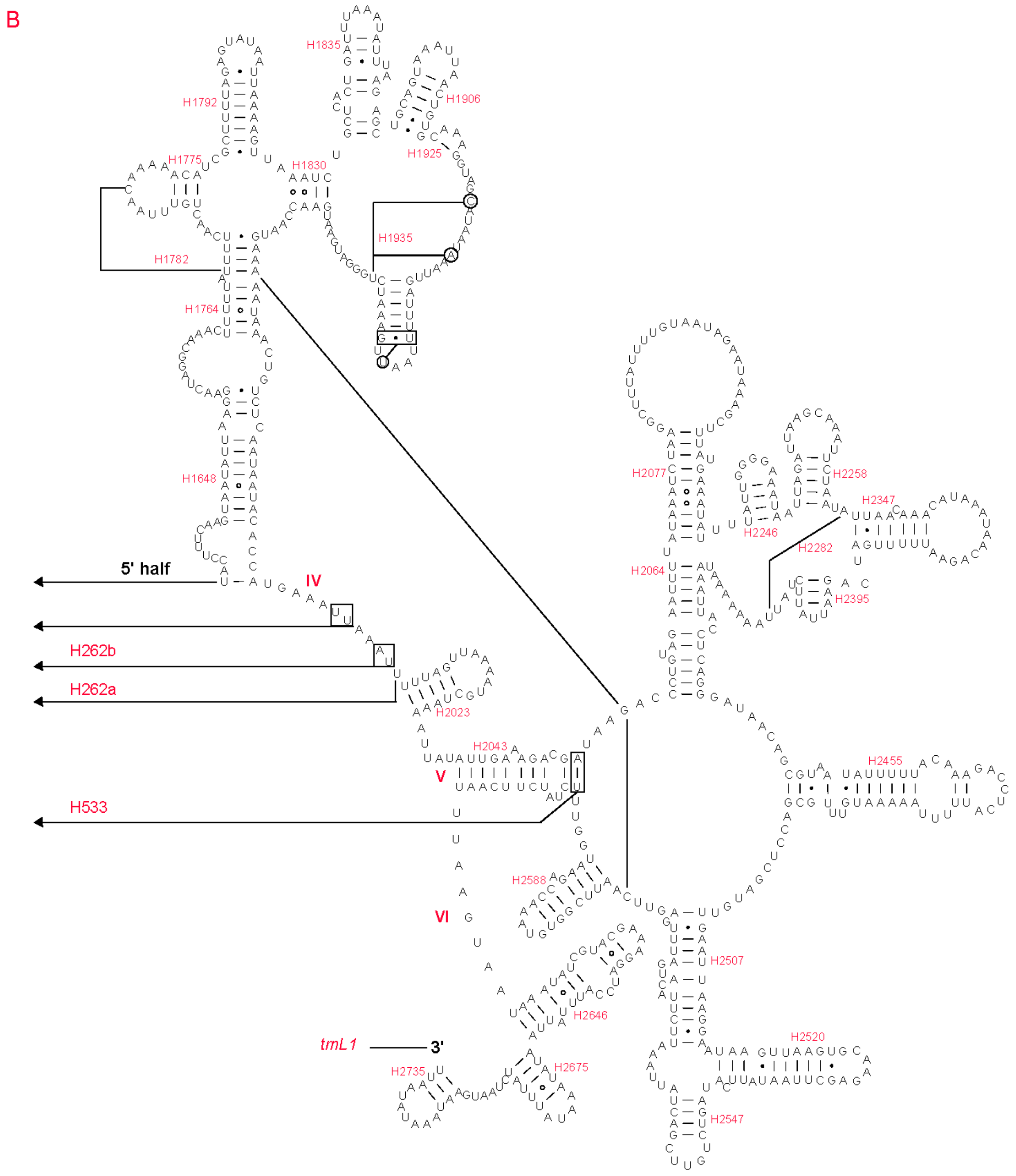

Figure S4. Predicted *rrnL* secondary structure in the *Agenioideus* mitochondrial genome. (**A**) 5′ half of *rrnL*; (**B**) 3′ half of *rrnL*. Symbols are as in Figures S1 and S3. I–VI shows the region of different domains.

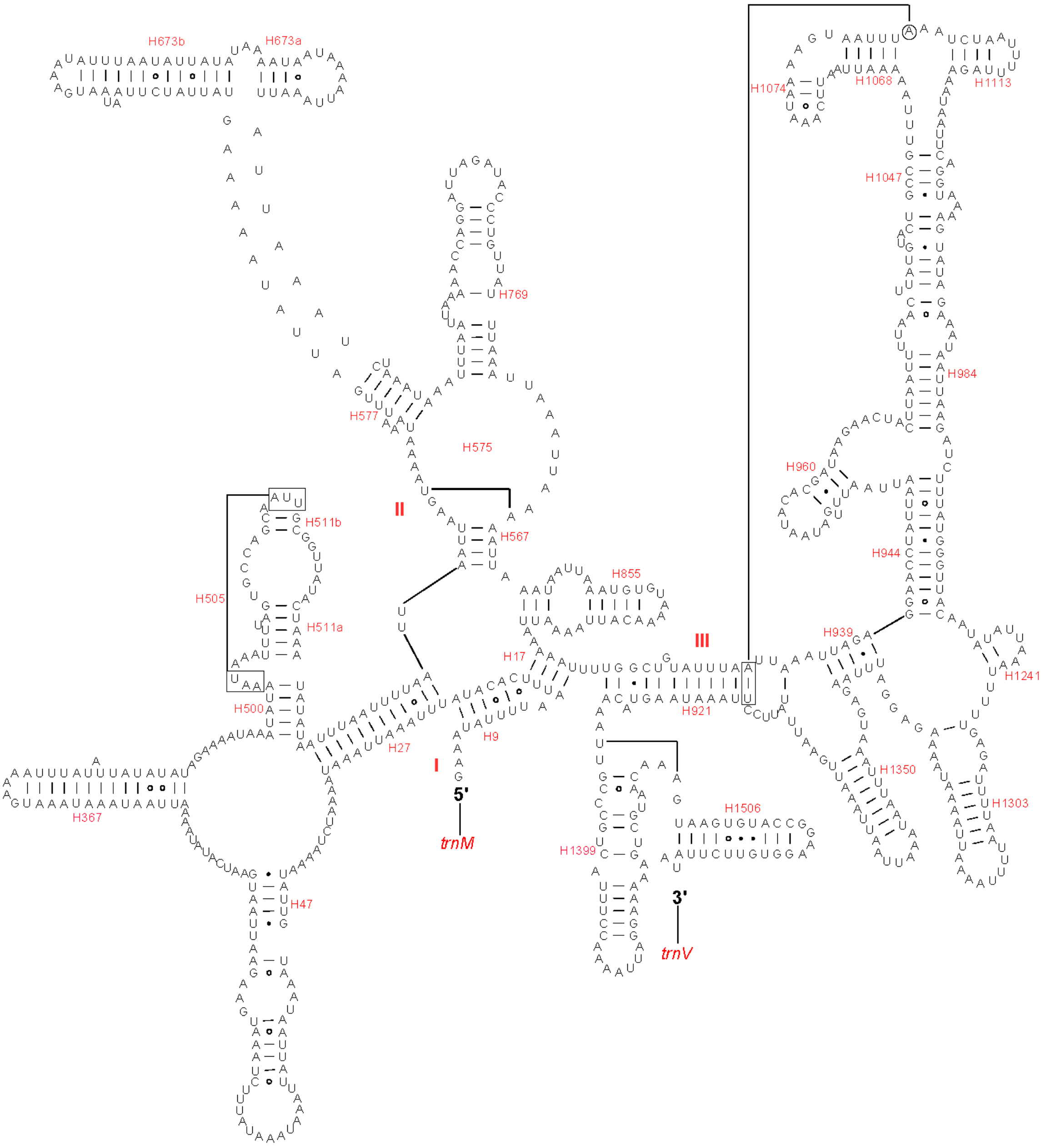

Figure S5. Predicted *rrnS* secondary structure in the *Auplopus* mitochondrial genome. Symbols are as in Figures S1 and S3. I–VI shows the region of different domains.

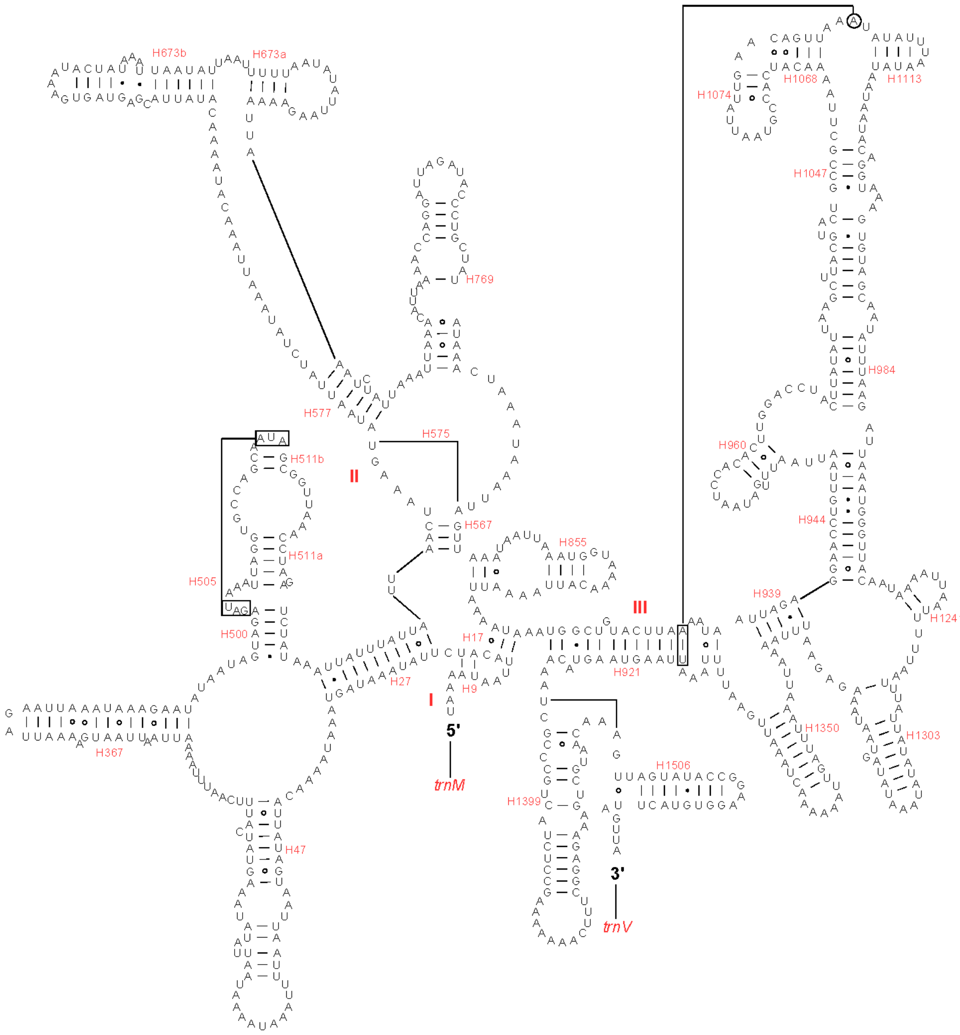

Figure S6. Predicted *rrnS* secondary structure in the *Agenioideus* mitochondrial genome. Symbols are as in Figures S1 and S3. I–VI shows the region of different domains.
